# Ambient Artificial Intelligence Scribes and Physician Financial Productivity

**DOI:** 10.1001/jamanetworkopen.2025.53233

**Published:** 2026-01-09

**Authors:** A Jay Holmgren, Cynthia L. Fenton, Robert Thombley, Hossein Soleimani, Rhiannon Croci, Orianna DeMasi, Maria E. Byron, Sara G. Murray, Julia R. Adler-Milstein, Jinoos Yazdany

**Affiliations:** 1 Department of Medicine, University of California San Francisco, San Francisco

## Abstract

This cohort study evaluates physician revenue, patient volumes, and claim denials among adopters and nonadopters of artificial intelligence–based clinical documentation tools in one health system.

## Introduction

Adoption of ambient artificial intelligence (AI) scribes, which generate clinical documentation from audio recordings and are associated with reduced documentation time and burnout,^1,2,3^ is increasing. Little is known regarding AI scribes and revenue changes. Without requirements to see more patients, increased relative value units (RVUs) or decreased claim denials may help mitigate the expense of AI scribes.^4^ Understanding their financial implications also informs policymakers of potential increases in health care spending. We examined whether ambient AI scribe adoption is associated with changes in RVUs, ambulatory visit volume, and claim denials.

## Methods

We extracted electronic health record (EHR) metadata at University of California San Francisco (UCSF) Health from January 1, 2023, through April 1, 2025, for all ambulatory visits completed by an attending physician. We excluded encounters without a note, to which another author contributed a note, or for which multiple clinicians submitted charges from encounter-level analyses. The UCSF Institutional Review Board approved this cohort study and waived informed consent for secondary analysis of EHR data. We followed the STROBE reporting guideline.

Our primary independent variable was whether the physician had access to (adopter of) 1 of 2 commercial AI scribe tools at the time of the encounter. The preadoption period included all encounters before enrollment or first use of an AI scribe, and the postadoption period included all encounters after scribe adoption. UCSF Health offered 2018 attending physicians access to an AI scribe, starting August 7, 2023, with a broad rollout beginning March 4, 2024. Physicians selected when to use the tool. There were no additional productivity requirements for AI scribes, and results reflect physicians’ voluntary response.

We specified 2 outcomes at the encounter level (total RVUs billed and any payer denials) and 2 outcomes at the physician-week level (total RVUs and ambulatory encounters per week). Covariates included modality (in-person, telemedicine); new or established patient visit; encounter length; time-based billing; and patient race and ethnicity, gender identity, interpreter need, payer, and Charlson Comorbidity Index score over the year before the encounter.

We compared outcomes using multivariable ordinary least squares regression with a difference-in-differences framework. Models included physician fixed effects to control for unobserved time-invariant confounders, week fixed effects to control for secular changes, and covariates with robust SEs clustered at the physician level. Event study models assessed outcomes over time.

Two-sided *P* < .05 indicated statistical significance. Data analysis was performed with Stata 17.0 (StataCorp LLC).

## Results

Of the 1 908 160 ambulatory encounters in the sample, 1 202 734 (across 341 711 unique patients and 1565 physicians) met our encounter-level inclusion criteria. Among physicians, 698 (44.6%) were AI scribe adopters and 867 were nonadopters; 182 617 encounters (15.2%) were with adopters.

**Figure 1.**
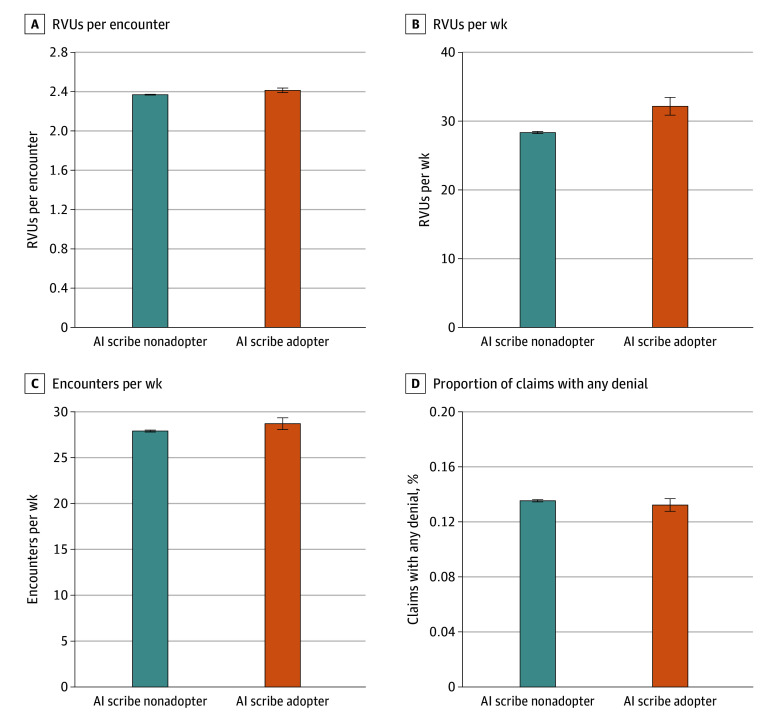
Association Between Physician Financial Productivity and Artificial Intelligence (AI) Scribe Adoption Results were calculated with difference-in-differences ordinary least squares regression models using 2-way fixed effects as well as time-varying controls for new- or established-patient visits, Charlson Comorbidity Index scores, modality, gender identity, payer, race and ethnicity, encounter length, time-based billing, and interpreter use, with robust SEs clustered at the physician level. Self-identified race and ethnicity were included in the study to control for potential differences in payment and care intensity across racial and ethnic groups. Error bars represent 95% CIs. RVU indicates relative value unit.

**Figure 2.**
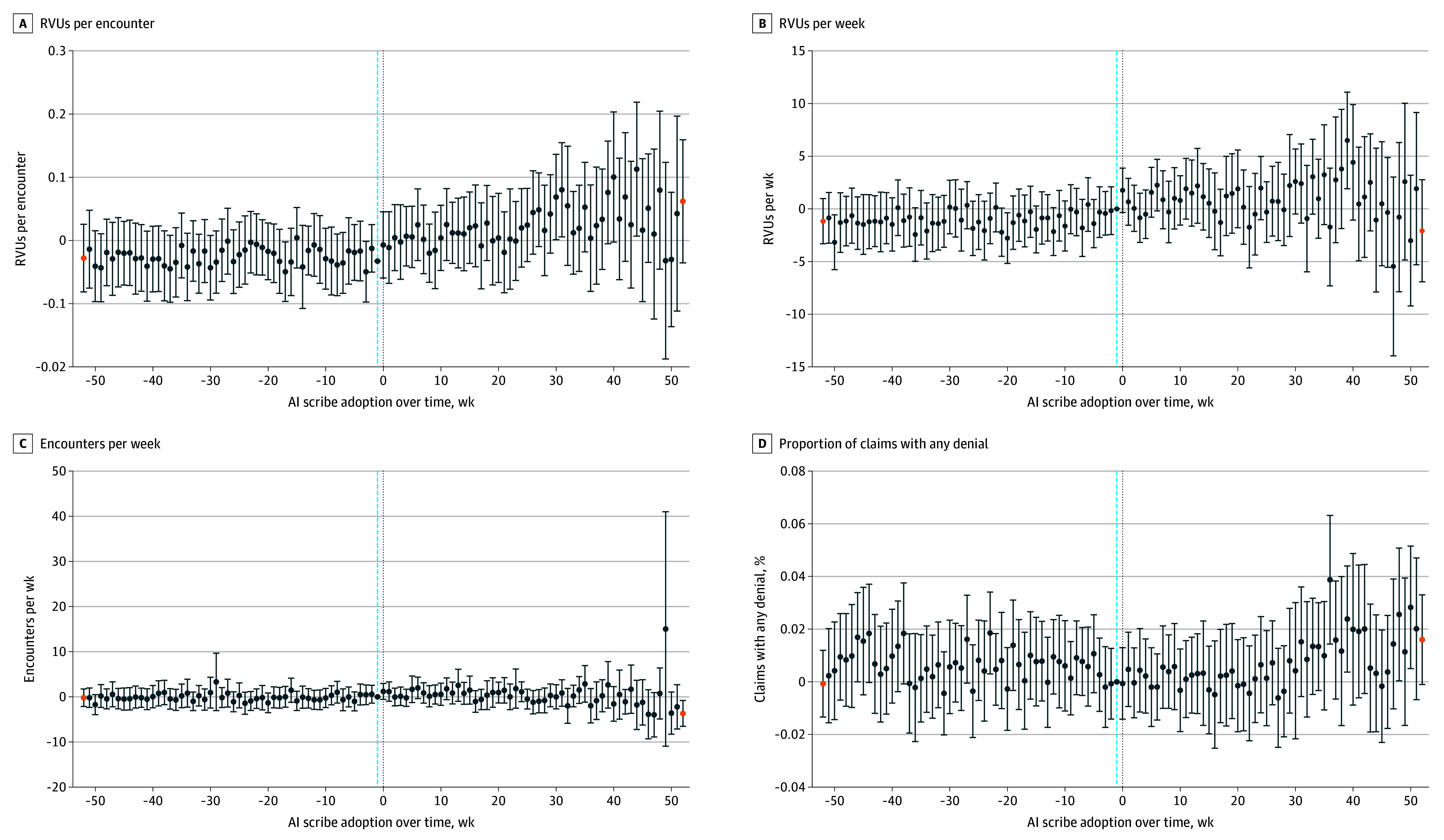
Outcome of Artificial Intelligence (AI) Scribe Adoption for Physician Financial Productivity Over Time Results were calculated with event study ordinary least squares regression models using 2-way fixed effects as well as time-varying controls for new- or established-patient visits, Charlson Comorbidity Index scores, modality, gender identity, payer, race, encounter length, time-based billing, and interpreter use, with robust SEs clustered at the physician level. The blue dashed line indicates the reference week (1 week before adoption), black dotted line indicates time 0 (first week of adoption), blue circles indicate the coefficient estimate, and orange circles indicate 52 weeks before and after adoption. Error bars represent 95% CIs. Wider CIs in later postadoption weeks reflect smaller sample sizes due to staggered adoption.

Encounters with adopters had 0.04 (95% CI, 0.02-0.07) greater RVUs per encounter (*P* = .001). Adopters had 1.81 (95% CI, 0.86-2.75) greater RVUs per week relative to nonadopters (*P* < .001). Adopters also had 0.80 (95% CI, 0.05-1.56) more encounters per week vs nonadopters (*P* = .04). There was no difference in proportion of claims with a denial (Figure 1). RVUs increased over time (Figure 2). Event study coefficients in the preadoption period were near 0, consistent with parallel preadoption patterns.

## Discussion

AI scribe adoption was associated with increases in RVUs and encounters per week, with no evidence of increased denials. These findings inform health systems on AI scribes’ return: 1.81 RVUs per week increase translates to $3044 annually per physician, using the 2025 Medicare Physician Fee Schedule. Study limitations include EHR metadata from 1 site, which may not capture the universe of confounders, and potential self-selection bias in early adopters. Further research should assess factors in AI scribe use, ascertain whether increased RVUs reflect more clinical services or accurate coding rather than upcoding, and strengthen causal association using randomization or other natural experiments.

## Notes

Supplement.
Data Sharing Statement
