# Supplementary material for: Ambient Artificial Intelligence Scribes and Physician Financial Productivity
Source: JAMA Netw Open. 2026 Jan 9;9(1):e2553233. doi: 10.1001/jamanetworkopen.2025.53233 (PMC12789954; doi:10.1001/jamanetworkopen.2025.53233)
Supplement: Supplement. — Data Sharing Statement [file jamanetwopen-e2553233-s001.pdf]

Data Sharing Statement

Holmgren. Ambient Artificial Intelligence Scribes and Physician Financial Productivity. *JAMA Netw Open*. Published January 09, 2026. doi:10.1001/jamanetworkopen.2025.53233

Data
Data available: No

Additional Information
Explanation for why data not available: Data includes PHI and cannot be shared outside of UCSF. Analytic code is available upon request.
